# Photodynamic Therapy in *Pythium insidiosum* – An In Vitro Study of the Correlation of Sensitizer Localization and Cell Death

**DOI:** 10.1371/journal.pone.0085431

**Published:** 2014-01-21

**Authors:** Layla Pires, Sandra de Moraes Gimenes Bosco, Maurício S. Baptista, Cristina Kurachi

**Affiliations:** 1 São Carlos Institute of Physics, University of São Paulo, São Carlos, São Paulo, Brazil; 2 Instituto de Biociências de Botucatu, UNESP Univ Estadual Paulista, Botucatu, São Paulo, Brazil; 3 Institute of Chemistry, University of São Paulo, São Paulo, São Paulo, Brazil; Royal College of Surgeons, Ireland

## Abstract

Pythiosis is an infectious disease caused by *Pythium insidiosum,* a fungus-like organism. Due to the lack of ergosterol on its cell membrane, antibiotic therapy is ineffective. The conventional treatment is surgery, but lesion recurrence is frequent, requiring several resections or limb amputation. Photodynamic therapy uses photo-activation of drugs and has the potential to be an attractive alternative option. The *in vitro* PDT response on the growing of *Pythium insidiosum* culture was investigated using three distinct photosensitizers: methylene blue, Photogem, and Photodithazine. The photosensitizer distribution in cell structures and the PDT response for incubation times of 30, 60, and 120 minutes were evaluated. Methylene blue did not penetrate in the pathogen's cell and consequently there was no PDT inactivation. Photogem showed heterogenous distribution in the hyphal structure with small concentration inside the cells. Porphyrin-PDT response was heterogenous, death and live cells were observed in the treated culture. After 48 hours, hyphae regrowth was observed. Photodithazine showed more homogenous distribution inside the cell and with the specific intracellular localization dependent on incubation time. Photodithazine first accumulates in intracellular vacuoles, and at incubation times of one hour, it is located at all cell membranes. Higher inhibition of the growing rates was achieved with Photodithazine -PDT, over 98%. Our results showed that the photosensitizers that cross more efficiently the *Pythium insidiosum* membranes are able to cause extensive damage to the organism under illumination and therefore, are the best options for clinical treatment.

## Introduction

*Pythium insidiosum* is a fungus-like organism that in contrary to other species of *Pythium*, shows pathogenicity to several animal classes. Its life cycle was described by Mendoza et al., who reported that, during zoosporogenesis, the hyphae starts to differentiate, and a plug is formed at the base of the apex. The biflagellated zoospores are produced inside a vesicle-like structure that cleaves when the zoospore numbers are extensively high. The motile zoospores transpose and break the vesicle wall, being released in water. The zoospores can attach to a plant or animal tissue and begin an encystment process, forming the germination tube [1].

Oomycetes differ from true fungus in many aspects as mitochondria with tubular cristae; Golgi bodies consisting of multiple flattened tanks and the presence of electrodense organelle with lamellar arrangement. Other important feature is the cell wall mainly composed of β-1,3- e β-1,6-glucans, cellulose and hidroxyprolin [2]. The absence of ergosterol in the plasma membrane is the reason why *Pythium insidiosum* response to antifungal agents is unsatisfactory, since the inhibition of ergosterol synthesis is the major mechanism of action of these drugs [2].

This cell wall structure decreases drug penetration and consequently, the pythiosis treatment [3]. This disease occurs in tropical and subtropical regions and it is characterized by granulomatous ulcerative lesions, mainly in cutaneous and subcutaneous tissues [4], and it may be life-threatening in some cases [5].

In Brazil, pythiosis cases were reported in horses, sheep [6], dogs [7], goal, calves [8], cattle and one case in human [9]. Pantanal is an endemic region, and possibly the highest worldwide incidence area [10]. In USA more than hundred cases of canine pythiosis were described [7]. Thailand is endemic region of human pythiosis in ocular, vascular and cutaneous forms with high rates of mortality and morbidity [11].

The conventional treatment for the cutaneous form is aggressive surgery and limb amputation, but it cannot be indicated to all anatomical sites, due to the requirement of a large margin resection. The difficulty on detecting the hyphae infection in the tissue results in high recurrence rates [12], [13]. Immunotherapy showed some positive results for equine, but this is not observed in all treated animals, or in other species. Associations of surgery, immunotherapy and antifungal therapy are also used but results are still not completely effective [14]. The lack of an efficient treatment and the increase in the number of cases and affected species make the development of new therapeutics options for pythiosis extremely relevant.

Photodynamic therapy (PDT) is mainly indicated for the treatment of cancer lesions, but other cutaneous diseases have been also targets, as psoarisis, herpes, and infections [15]–[19]. It is based on the interaction between a dye and light, at a specific wavelength, in the presence of oxygen to cause cell death. Under irradiation, the dye, called photosensitizer (PS), reacts with the molecular oxygen producing reactive oxygen species (ROS) that are highly toxic for cells [20]. The presence of the PS linked to cell structures is essential for an effective photodynamic reaction. PS distribution inside the cell is also related to the internalization process and to the damage caused by the oxygen pathways. Since the lifetime of the ROS in biological systems is around 0.04 µs, its reaction length is extremely short, only cellular structures close to the excited PS molecule will be harmed by PDT [20]–[22]. In this way, the PS subcellular localization and concentration present information of the overall phototoxicity and cell death mechanism. The PS-cell interaction depends on the PS chemical structure, mainly on its characteristics of electron charges, hydrophobicity and lipophilicity.

**Figure 1:**
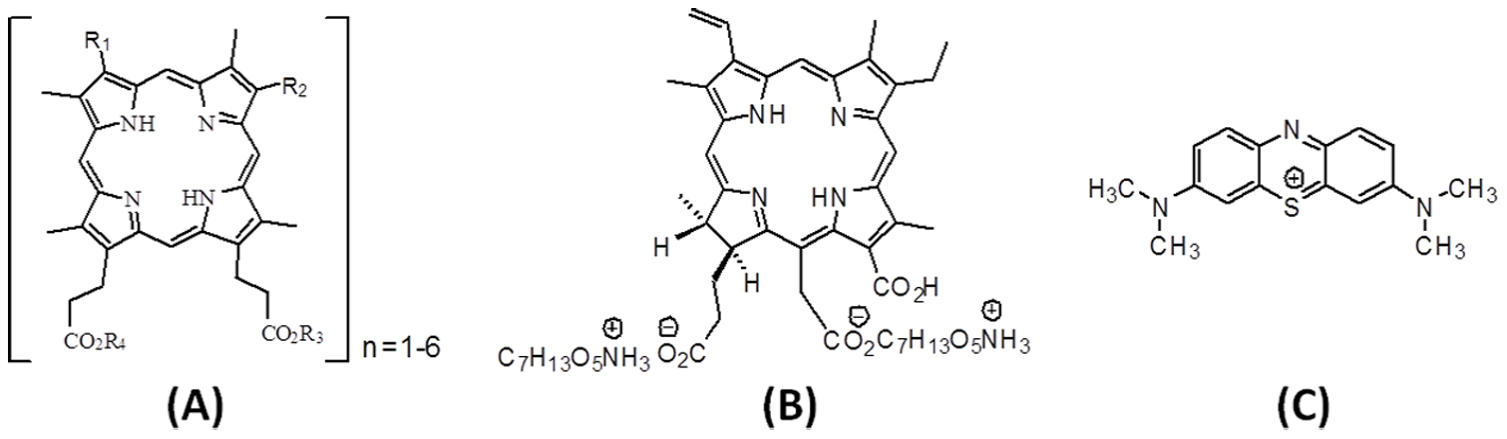
Molecular structures of Photogem (A), Photodithazine (B) and MB (C).

The photosensitizers used in this study are from three different classes: porphyrin, chlorine and phenotiazinium. Photogem® is a haematoderivative porphyrin (HpD) of the first generation of photosensitizers (Figure 1-A). HpD is a mixture of monomer, dimer and oligomers compounds [23]. Photogem has an intense absorbance in violet region of the spectrum and weaker absorbance in the red spectrum. Major clinical disadvantage is the long skin photosensitivity. The absorption band at 630 nm shows a weak molar extinction coefficient of 1170 M^−1^cm^−1^ [24]. The reduction of a pyrrole double bond on the porphyrin periphery gives the chlorine core. Chlorine e6 is derived from oxidation of chlorophyll a and has a high absorption at 654 nm with a molar extinction of 40000 M^−1^cm^−1^ [24]. Photodithazine® is a commercial water soluble glucosamine salt of chlorine(e6) (Figure 1-B).

Methylene blue (MB) is a phenotiazinium dye, widely used for microorganism inactivation [25]. It presents low toxicity towards humans and a high absorption at 656 nm (Figure 1-C).

Due to the increasing number of microbial resistance strains to antibiotic therapy, PDT may constitute a new strategy to inactivate them. The PDT response for microorganism control, as well as the indicated protocol for each microorganism type must be determined. In this study, the efficacy of PDT for the growth control of *Pythium insidiosum* was investigated and the photodynamic response correlated to the sensitizer localization.

## Materials and Methods

### Pythium isolate and culture

It was used an isolate from Professor Sandra de Moraes Gimenes Bosco collection obtained from a horse at the School of Veterinary Medicine and Animal Science at Universidade Estadual Paulista (Botucatu, SP, Brazil). We choose to evaluate the effect of PDT on fresh isolate from a naturally infected animal seeking a clinical application of the technique. Cell cultures were maintained on Sabouraud dextrose agar (SDA, Difco, USA), incubated at 37°C, and recultured weekly.

### Zoosporogenesis

The isolate was cultured in SDA for 24 hours, and the medium nutrients were gradually reduced, SDA 4%, SDA 2% and then agar 2%. When the isolate was cultured in agar, sterile grass fragments were added to induce the plant parasitism. Then, the infected grass was transferred to the induction medium as described previously by Santurio et al., 2003 [26]. The zoospores were counted using Neubauer chamber.

### Photosensitizers

Three photosensitizers were used in this study, haemato porphyrin derivative (Photogem®, Russia), glucosamine salt of chlorine (e6) (Photodithazine®, Russia) and MB (Sigma Aldrich®) in distilled water solutions of 10 mg/mL, 0.7 mg/mL, and 100 µg/mL, respectively.

### Light source

Light emitting diode (LED)-based devices were used, one with emission around 530 nm for haematoderivative porphyrin assays, and another system emitting around 660 nm for glucosamine salt of chlorine(e6) and MB assays. Both light sources were set at irradiance of 65 mW/cm^2^. The fluences evaluated are 30, 50 and 70 J/cm^2^.

### Survival fraction assays

The PDT effect was evaluated on *Pythium insidiosum*'*s* zoospores following the CLSI M38-A2 microdilution techniques for filamentous fungi. One milliliter with ten thousand zoospores in RPMI medium without bovine fetal serum and fenol, was cultured in 24-well plates. Ten microlliters of the each sensitizer were added and thirty minutes after, the irradiation was performed. Each condition was repeated three times and the complete experiment was also performed three times. After PDT, the zoospore solution was cultured in 10-fold serial dilutions in Sabouraud Dextrose Agar and cultured at 37°C. Twenty four hours after the culture, the colony forming units were evaluated.

### Inhibition rate assays

The PDT effect was also evaluated on the hyphae growth of *Pythium insidiosum* based on the analysis of its cell wall and membranes characteristics. For experimental purposes, standardized fragments of 5mm diameter were obtained from the borders of the culture and sub-cultured onto SDA.

#### PDT

Standardized fragments were cultured on SDA, and 10 µl of the sensitizer solution was added to the fragment. Three incubation times of 30, 60 and 120 minutes were investigated. After the incubation time, irradiation was performed with a delivered fluence of 70 J/cm^2^. Five replicates were performed for each assay.

#### Inhibition growth rate

Cultures were imaged 48 h after each treatment. The growth area was measured using ImageJ® and the inhibition rate was calculated (equation 1).
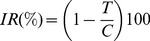
(1)Where T represents the growth area of treatment group and C the growth area of control group (no treatment). Statistical analyses were performed using ANOVA and Kruskal-Wallis with significance of 95%.

### Sensitizer cellular distribution

*P. insidiosum* was cultured in Sabouraud Dextrose Broth (SDB) for 24 hours. After this period, it was washed five times to reduce the medium present in the cells. The fragment was then immersed in photosensitizer solution at concentration of 150 µg/mL. After incubation time of 30, 60, or 120 minutes, the fragments were washed with distilled water and imaged at confocal microscope (LSM780, Zeiss, Germany) in a coverslip. The samples, sensitized with porphyrin and chlorine, were illuminated at 405 nm and the signal was captured in two channels, one for acquisition of the microorganism natural fluorescence (450–600 nm), and the other, of the photosensitzer fluorescence (600–700 nm). For the samples sensitized with MB, the illumination was performed at 594 nm, and the emission detection at 450–600 nm, and 600–700 nm.

### Monitoring of the PDT effect at confocal microscope

*P. insidiosum* was cultured in SDB for 24 hours, washed, and immersed in photosensitizer solutions at concentration of 150 µg/mL. After the incubation time, the sample was washed with distilled water, irradiated for 10 minutes, delivering a total fluence of 30 J/cm^2^. Just after PDT illumination, the samples were imaged at confocal microscope, using the same parameters described before.

## Results

### Survival fraction

**Figure 2:**
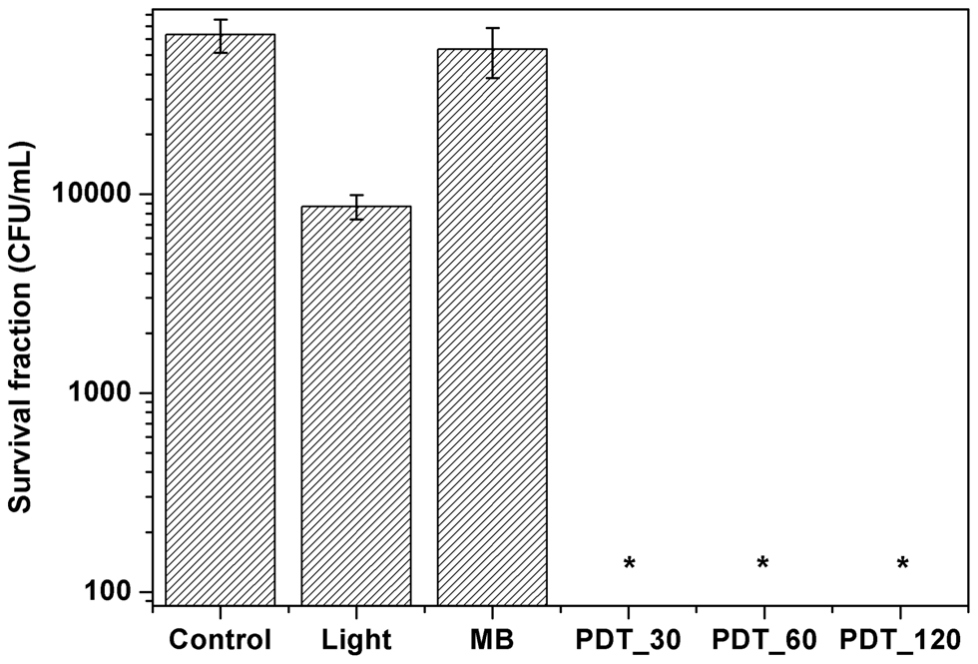
Pythium insidiosum's zoospores survival fraction after PDT treatment using methylene blue at 100 µg/mL. MB refers to the zoospores incubated only in the dye for 120/cm^2^. 30, 60 and 120 represents the zoospores incubated with these different times and then irradiated with 70 J/cm^2^.

**Figure 3:**
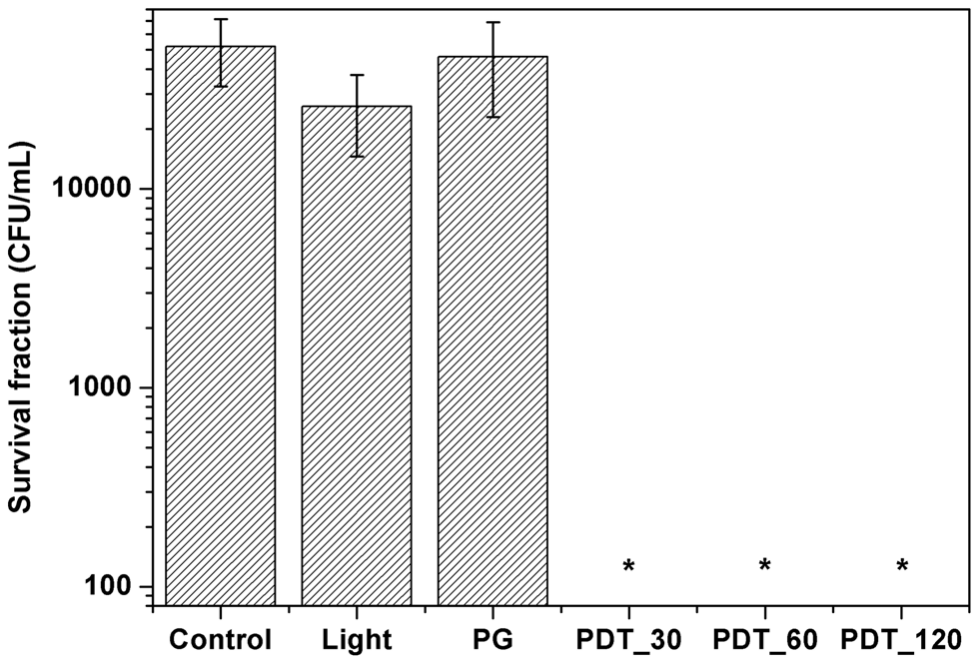
Survival fraction for the zoospores treated with photodynamic therapy using Photogem. Photogem (10 mg/mL) and light (70 J/cm^2^) group did not show statistically significant difference when compared to control group. All PDT protocols inactivated the zoospore form.

**Figure 4:**
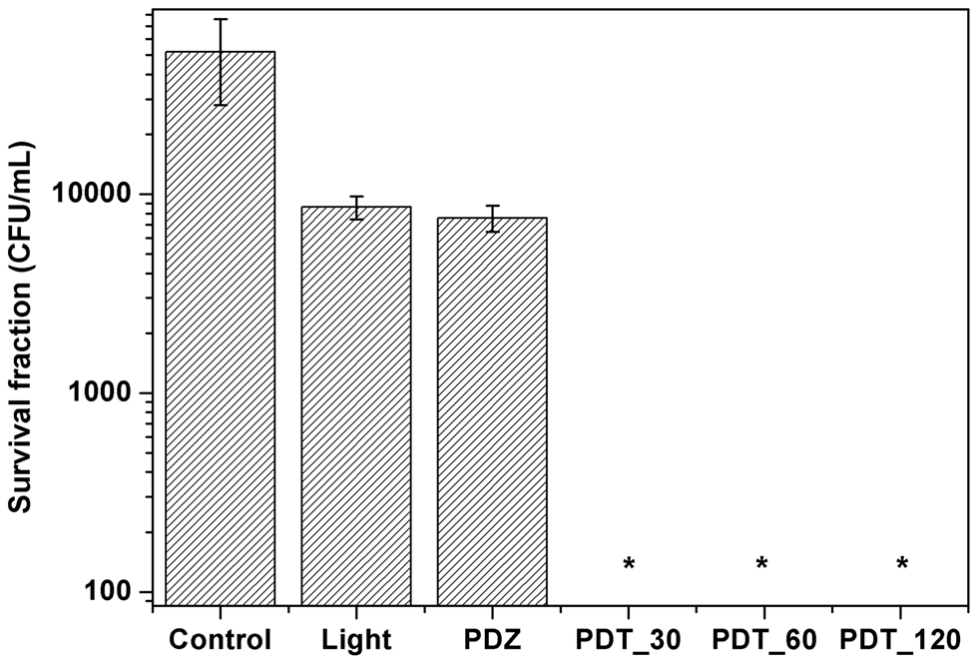
Photodynamic therapy with Photodithazine on *Pythium insidiosum's* zoospores. Photodithazine (1.3 mg/mL) and light (70 J/cm^2^) group did not show statistically significant difference when compared to control. PDT showed high effect on the pathogen inactivation.

Figure 2 shows the survival fraction for the control, light and methylene blue groups for different incubation times. The treatment groups of light and methylene blue showed only a small reduction that was not statistical significantly. On the other hand, in the PDT group none colony was observed, the result is statistically significant when compared to control group. The same behavior was observed for the other photosensitizers, Photogem (Figure 3) and Photodithazine (Figure 4).

### Inhibition rate

**Figure 5:**
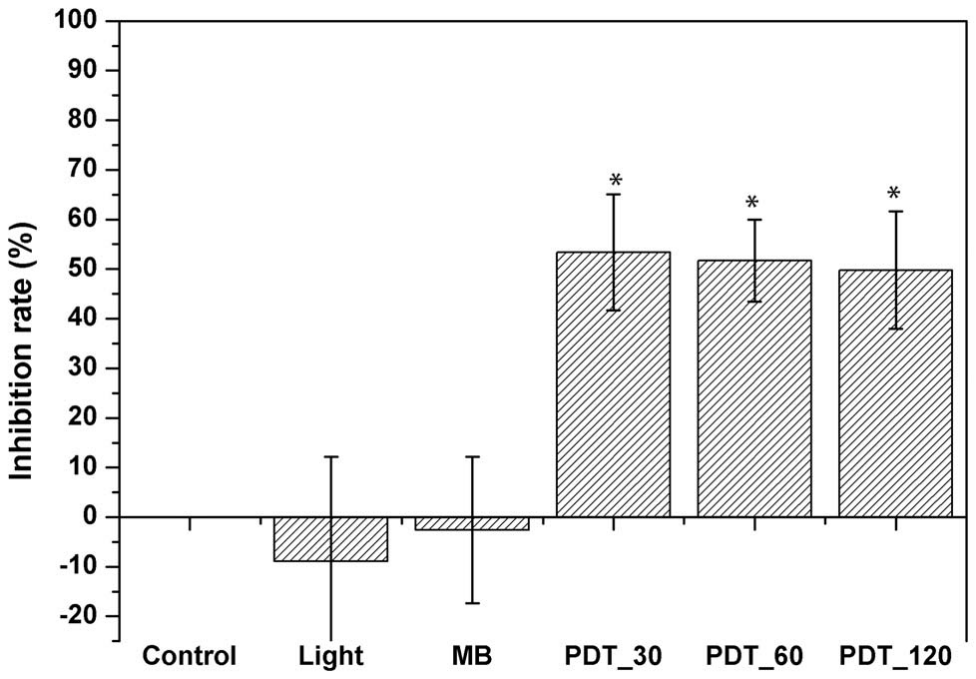
Inhibition rates after PDT treatment using methylene blue at 100 µg/mL. MB refers to the group treated only with the dye for 120/cm^2^. 30, 60 and 120 refer to incubation times in minutes. Statistical significance was observed between treated and control groups.

Figure 5 shows the inhibition rate for methylene blue with different incubation times. All investigated protocols showed inhibition rates higher than 50%, but culture regrowth was observed within 7 days after treatment. Statistical significance was observed for all PDT groups when compared to control group.

**Figure 6:**
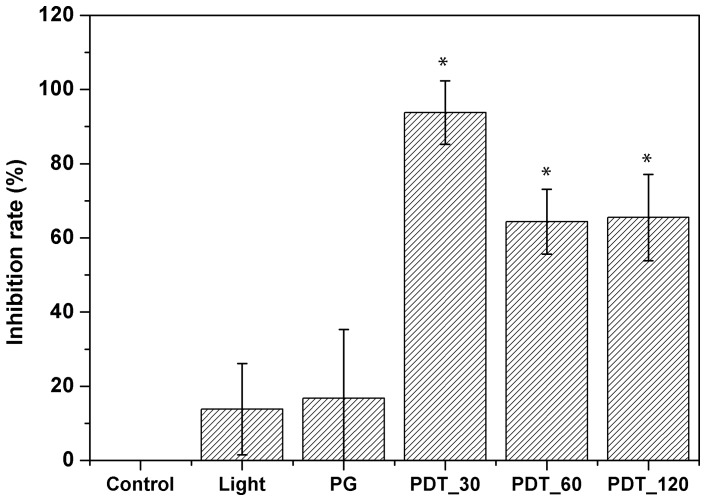
Inhibition rates for PDT treatment using Photogem at 10/mL. PG refers to Photogem group with no irradiation and incubation time of 120_30, PDT_60 and PDT_120 refer to PDT groups and incubation times of 30, 60 and 120 minutes, respectively. Light and PG groups did not show statistical difference with the control group. On the other hand, all PDT protocols evaluated were statistically different to the control group.

Photogem was more effective at 30 minutes of incubation. Increasing porphyrin incubation time resulted in a decrease of the inhibition rate for *Pythium insidiosum*. Photogem dark toxicity and light groups, did not show statistically difference on culture growth when compared to control (no treatment) group. All PDT protocols showed statistical difference to the control group (Figure 6).

**Figure 7:**
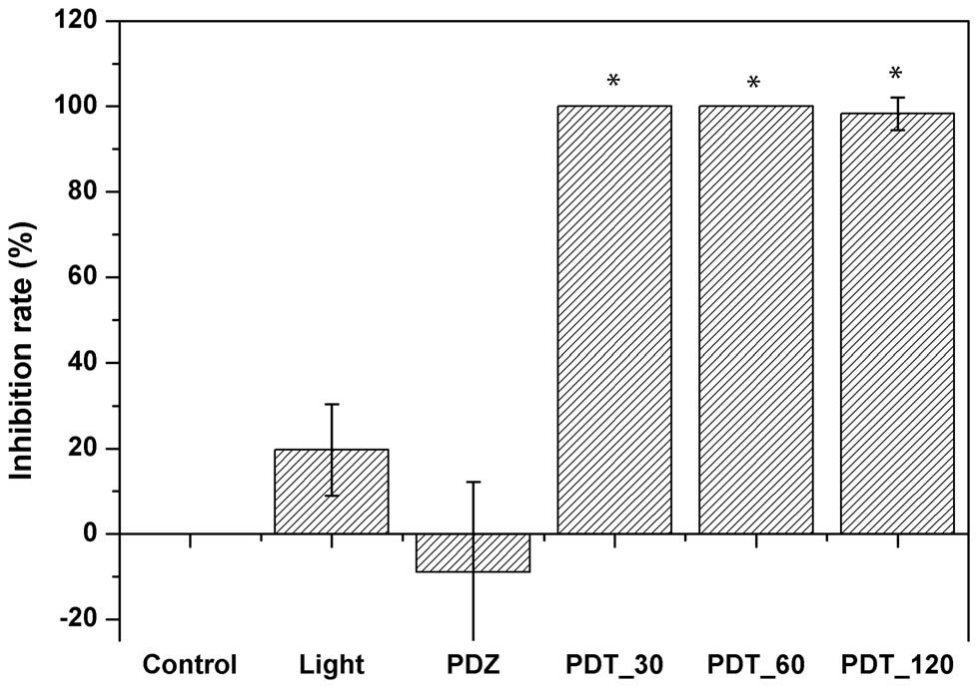
Inhibition rates for PDT using Photodithazine at 0.7 µg/mL. PDZ refers to Photodithazine group and incubation time of 120 minutes. Light is the irradiated group at 70/cm^2^. 30, 60 and 120 refers to incubation times in minutes. All PDT protocols showed statistical difference to the control group. Comparison between light and dye groups, alone, did not show statistical difference to the control group.

Photodithazine showed 100% of inhibition for 30 and 60 minutes of incubation until four weeks after treatment. For 120 minutes, only one of five fragments showed hyphae growth after PDT, after 48 hours of treatment. This dye showed the better inhibition results for all incubation times. All protocols showed statistical difference when compared to the control group, indicating the response of the pathogen to PDT. Chlorine and light alone groups did not show statistical difference to the control group (Figure 7).

These results may be explained by the pathogen's growth during the photosensitizer incubation time. In the first 30 minutes, the photosensitizer was available for a higher number of hyphae, resulting in a more effective PDT. The increasing in incubation time decreases the available photosensitizer molecules for young cells, and so the PDT effect. This fact was not observed for methylene blue due to the low interaction between this molecule and the pathogen. Chlorine showed a high inhibition rate even at longer incubation times, which was not observed for porphyrin. This fact may be explained by a possible mechanism of distribution of the chlorine to the daughter cells.

### Cell distribution of the photosensitizers

#### Pythium insidiosum

**Figure 8:**
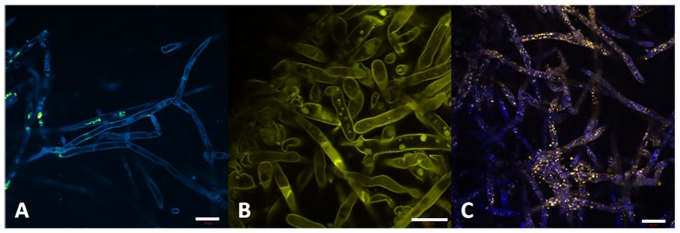
*P. insidiosum* autofluorescence (A); safranine dye showing the presence of cellulose in the cell wall and in a specific organelle (B); acriflavin dye marking cell nuclei (C).

Autofluorescence of the pathogen is characterized by emission at the blue-green region (410–580 nm). One can see the cell wall well-defined and a cylindrical morphology of the hyphae (Figure 8-A). In the Figure 5-B the pathogen was marked with safranine, evidencing the cellulose present in cell wall and some organelle. Nuclei were also target with acriflavin (Figure 8-C).

#### MB (Figure 9)

**Figure 9:**
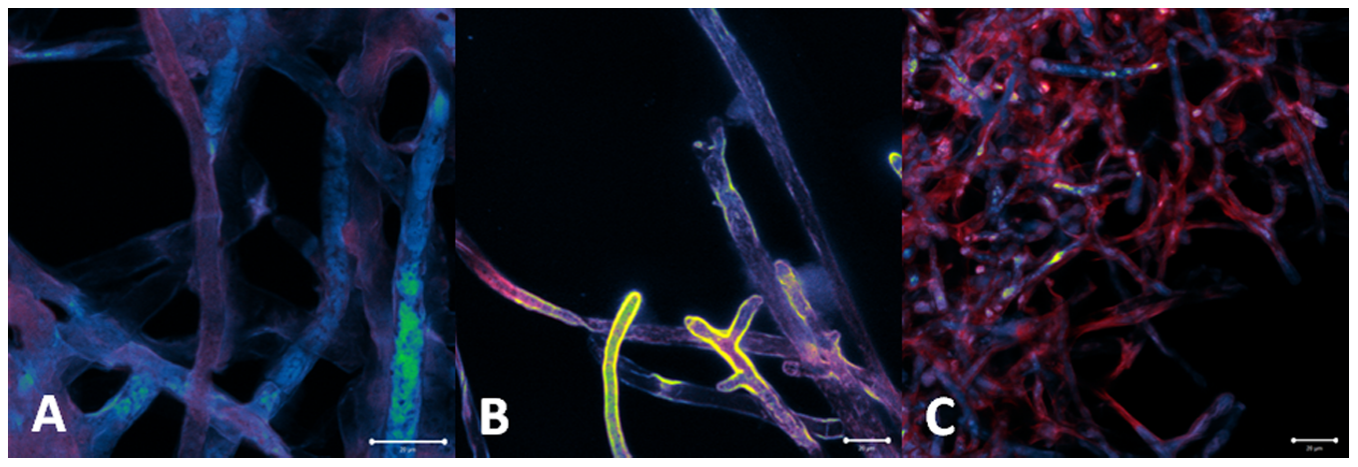
MB incubation for 30 (A), 60 (B) and 120 minutes (C) in concentration of 150 µg/mL. Large and cylindrical hyphae morphology is evident. No sensitizer (red fluorescence) is visualized inside the cells, suggesting dye localization only at hyphae surface.

For all incubation times, no dye molecules were observed inside the cells, only at the surface. This sensitizer probably does not bind to any membrane component, nor penetrate in the cell, being easily washed out from the samples.

#### Photogem (Figure 10)

**Figure 10:**
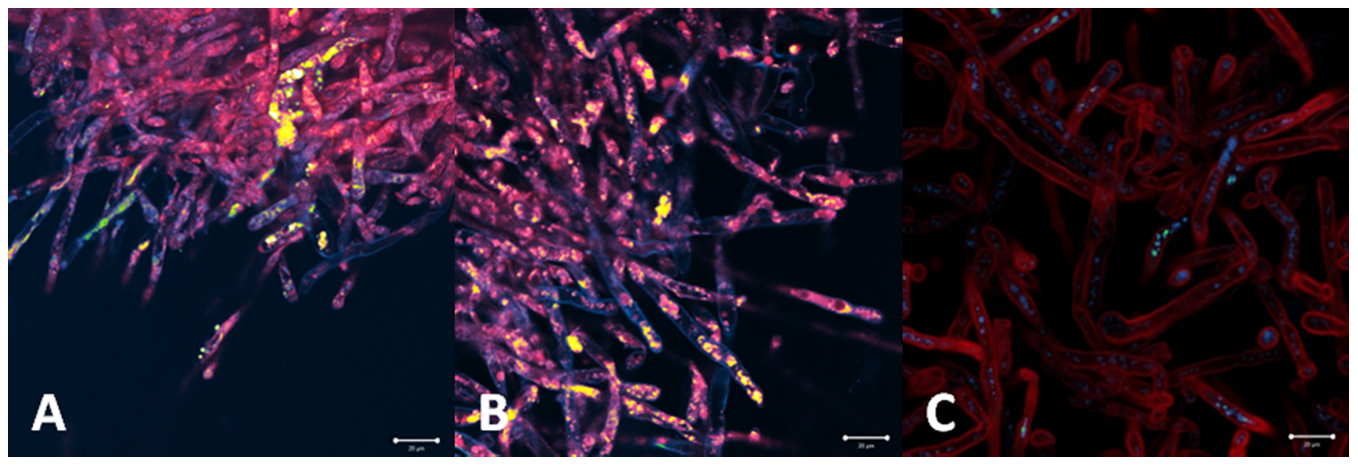
Porphyrin incubation for 30 (A), 60 (B) and 120 minutes (C) in concentration of 150 µg/mL. Morphology is preserved. Porphyrin (red) is initially present at the cell surface, and then it starts to be distributed in cell membrane.

For incubation time of 30 minutes, the sensitizer was distributed at the pathogen surface. For 60 and 120 minutes the porphyrin molecules were observed localized in cytoplasm and organelle membranes. Besides the intracellular presence, its distribution was heterogeneous. This non-uniform distribution inside the cell and among the whole hyphae culture, may justify the partial and less effective PDT effect.

#### Photodithazine (Figure 11)

**Figure 11:**
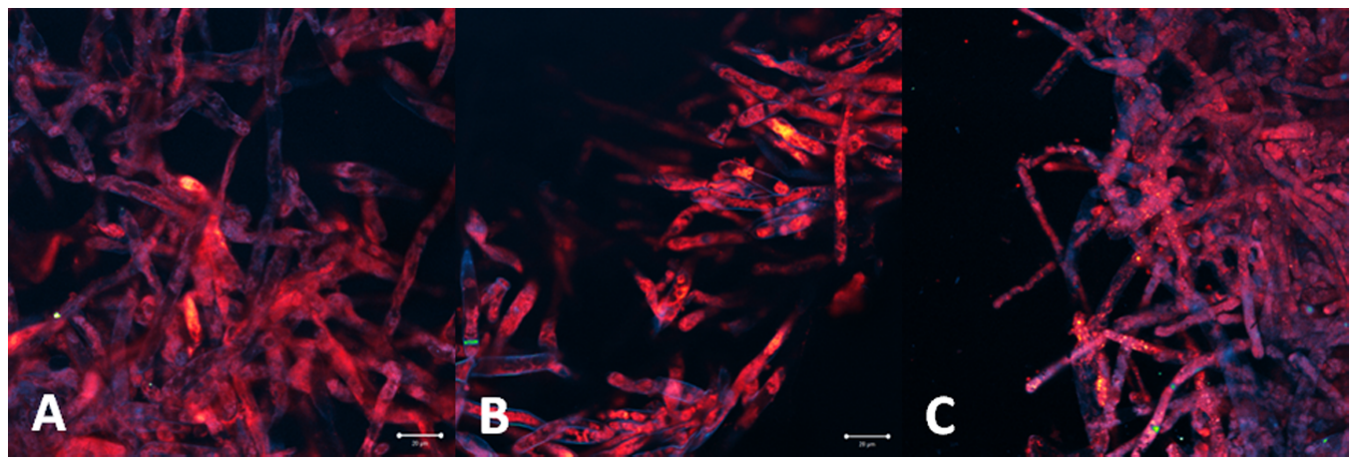
Chlorine incubation for 30 (A), 60 (B) and 120 minutes (C) in concentration of 150 µg/mL. Pathology morphology is preserved. Chlorine (red) is already present inside the cell surface after 30 minutes, and its distribution to all membranes is observed for longer incubation times.

At 30 minutes of incubation time, the sensitizer molecules were present in specific cylindrical organelles diffusely distributed inside the cells. The increase of the incubation time resulted in a higher distribution of the sensitizer. After 60 and 120 minutes of incubation, the photosensitizer was more uniformly distributed inside and among the cells of the sample, especially at organelle membranes. Differently from the heterogenous distribution observed for porphyrin, the chlorine was homogenously distributed in the hyphae.

### PDT response

#### MB

**Figure 12:**
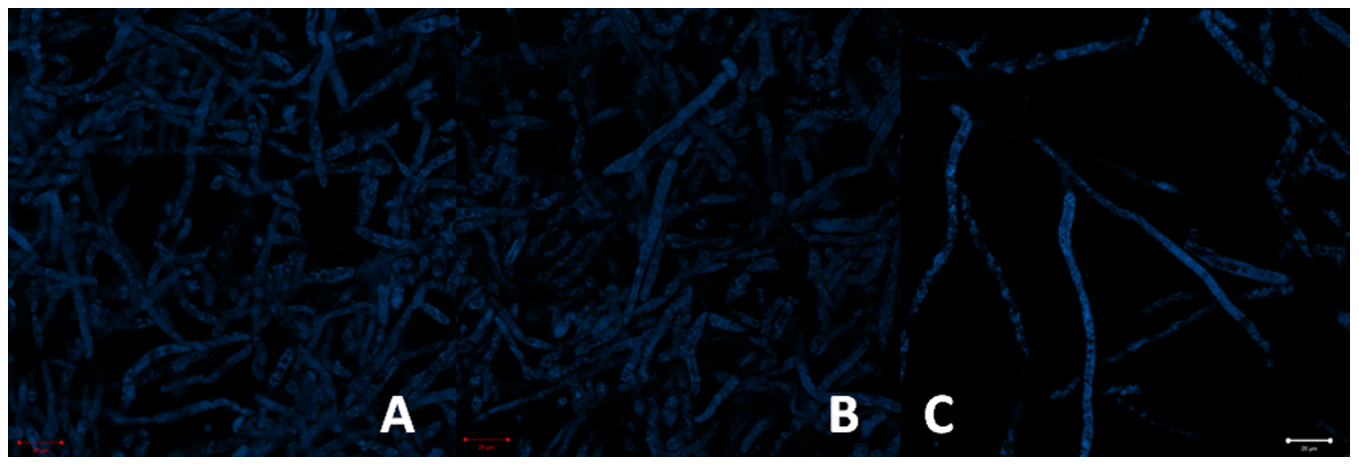
Preservation of the hyphae morphology indicated the PDT effect for all incubation time: 30 (A), 60 (B) and 120 minutes (C). It shows that PDT with methylene blue (100 µg/mL) was not effective to the pathogen.

The morphology integrity of the hyphae of *Pythium insidiosum* was preserved after PDT. No cellular damage was observed for all investigated parameters (Figure 12).

#### Photogem

**Figure 13:**
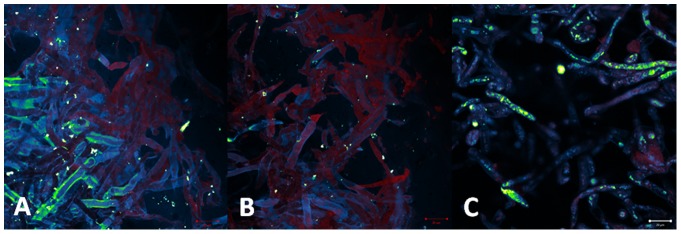
The PDT response observed was as heterogeneous as the porphyrin distribution. One can observe maintenance of cylindrical hyphae with areas of absence of fluorescence for 30 (A), 60(B) and 120 minutes (C) of incubation with porphyrin in concentration 10 mg/mL. This means that some hyphae was inactivated but others not.

The hyphae culture treated with incubation time of 30 minutes presented both inactivated and non-treated cells. Inactivated cells showed of the lack of the normal cylindrical morphology and presence of amorphous material. The partial cellular inactivation correlates with the heterogeneous sensitizer distribution observed for this incubation time. No improved PDT response was observed at the samples treated with higher incubation times (Figure 13).

#### Photodithazine

**Figure 14:**
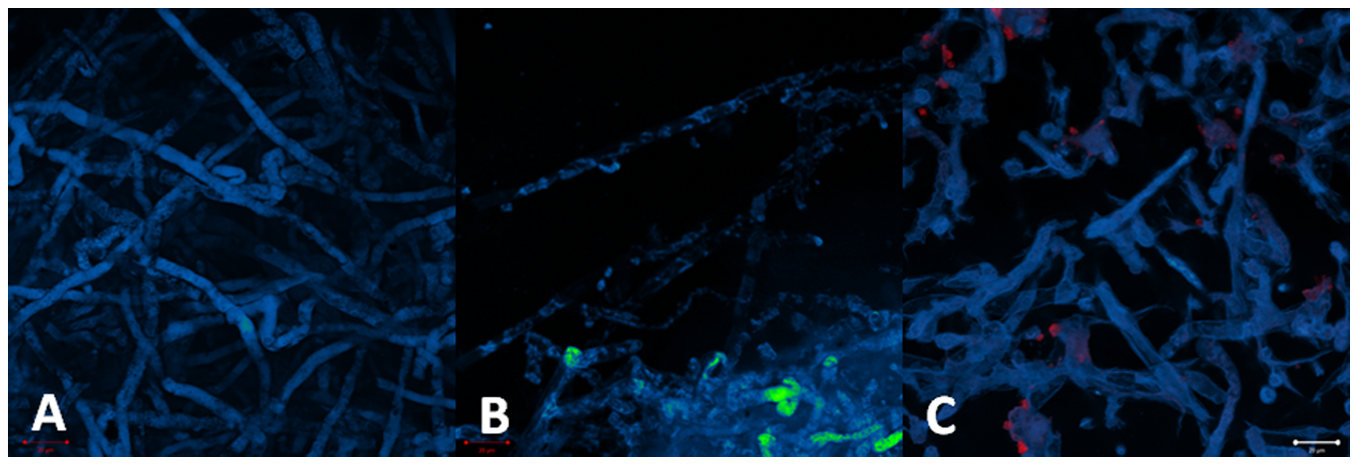
PDT response with chlorine in concentration of 0.7µg/mL can be observed by the cell rupture and cell material leakage. For 60 (B) and 120 minutes (C), when the dye is localized at cell membranes, the effect was more evident when compared with the incubation time of 30 minutes (A) when the dye is localized in a specific intracellular structure.

The fluorescence images of the hyphae samples treated with incubation time of 30 minutes showed the decreased autofluorescence matching the organelle localization with the higher sensitizer concentration. This evidence corroborates with the local PDT response mechanism. For longer incubation times, cellular damage is more evident with lack of cylindrical morphology, deposit of amorphous material and membrane rupture. PDT response with 60 and 120 minutes incubation times showed more homogenous damage to whole hypahe culture, result correlated to the observed homogenous distribution of the sensitizer (Figure 14).

## Discussion

Conventional treatments for pythiosis are antibiotic therapy and surgical resection. *In vitro* and *in vivo* responses of the antibiotic therapy reported in the literature are controversy, especially because ergosterol is the main target of the available drugs and it is absent at the *P. insidiosum* wall. Surgical resection presents high recurrence rates, due to the difficulty on the lesion extension. Also, depending on lesion size and location, surgery is not indicated. Based on the inefficacy of available methods, the development of a new treatment is mandatory. Photodynamic therapy has been reported as an alternative option for the local treatment of infections at the oral cavity, skin, among others tissues [27]–[29].

It is also reported that photodynamic therapy effect on planktonic microorganisms is higher when compared to the result on its biofilm. This fact was also observed when comparing the PDT effect on the zoospore and hyphae form. Zoospores are unicellular, ovoid and present flagella. At isolated cells, the dye is easily diffused,making the treatment more efficient. Moreover, the structural form of the zoospores is less resistant to PDT action. On the other hand, the complexity of the hyphal growth, in addition to the cell wall and inner membranes of the *Pythium insidiosum* cell, and the intricate net of hyphae, represent a tough barrier for drug diffusion. PDT inactivated the zoospores in all protocols evaluated. The dye alone showed decreasing survival fraction with the incubation time for all evaluated sensitizers. These results were not observed for the hyphae form.

Photosensitizers evaluated in this study showed three different interactions with *Pythium insidiosum* hyphae. MB did not penetrate into the cell, nor bind to surface structures. This may be explained by its molecular structure and electronic charge. MB is a cationic dye widely used for microorganism inactivation, especially for Gram+ bacteria. Although some studies show MB penetration in microorganism cells, this was not observed in *Pythium insidiosum*. The lack of interaction between this dye and the oomycete was evidenced with the fluorescence images with no presence of MB after washing the samples, and the maintenance of the cell integrity observed after PDT treatment. MB is positively charged and possibly is retained at lipopolysaccharide extracellular structures. This result was also proved at the inhibition rate assays, where no important growth control was achieved for different incubation times.

Photogem is a haematoderivative porphyrin compound that in water solution has monomer, dimer, and oligomer molecules. This sensitizer is widely used for cancer treatment, and also for microbiological control. The porphyrin penetrated into the pathogen cell and concentrated in cytoplasm and cell membranes. Although, this distribution was heterogeneous, some hyphae showed high concentration of porphyrin and in others, porphyrin was not visualized. The increase in incubation time did not result in an improved sensitizer distribution. Differently from the behavior observed for MB, where no cell interaction was observed, the porphyrin presented localization in intracellular structures. This difference may be explained by sensitizer charge characteristics. In biological medium, porphyrins show negative charge that improves transmembrane transport and intracellular accumulation. Since PDT is a treatment based on the interaction between the dye, light and oxygen, a heterogeneous sensitizer distribution is not adequate, since it induce a partial microorganism inactivation, leaving some hyphae alive, which results in culture regrowth. This result was supported by the inhibition rate assays, where the samples treated with porphyrin that did not result in complete inactivation, showed a hyphae regrowth at 24 hours after the treatment.

Amphiphilic molecules are known to interact strongly with biological membranes, which usually lead to improved PDT action [30]. Photodithazine is a glucosamine salt of chlorine(e6), it is an amphiphilic molecule that in biological medium shows negative charge, like porphyrins. The kinetic study showed distinct cellular localization of the Photodithazine for different incubation times. After 30 minutes, chlorine was observed in specific intracellular organelles. At 60 and 120 minutes, the sensitizer was more homogenously distributed inside the cell, targeting cell membranes. After PDT illumination, the localized action of this technique, based on the morphological changes at the cellular sites of higher concentration, could be observed. When the sensitizer was only evident inside the cylindrical organelles, the PDT effect was observed only in these cellular structures. On the other hand, when the sensitizer was more localized at membranes, the lack of cylindrical morphology and cell rupture were evident after treatment. This photosensitizer showed to be the most effective one for *Pythium insidiosum* inactivation due to the higher cell penetration and concentration in membranes. This result was validated by the inhibition rate assays with 100% of inactivation for 30 and 60 minutes of incubation.

*Pythium insidiosum* has a very different cell wall, when comparing to other microorganisms and mammalian cells. The presence of cellulose and tridimensional well-organized structures makes drug penetration and action, a pharmaceutical challenge. To the best of our knowledge, this is the first study that shows the PDT response for this pathogen inactivation, based on the photosensitizer cellular distribution and the *in vitro* inhibition rate. The investigation of the correlation of the photosensitization parameters and PDT response is relevant to improve the understanding of PDT mechanisms, and the establishment of more effective PDT protocols for the treatment of pythiosis.

## Conclusion

PDT response on *Pythium insidiosum* inactivation was investigated using three photosensitizers and three incubation times. The best results of inhibition growth rate were obtained with chlorine and incubation time of 60 minutes, with a total inactivation. PDT response was well-correlated with the photosensitizer cellular distribution.
